# Plasmonic enhanced Cu_2_O-Au-BFO photocathodes for solar hydrogen production

**DOI:** 10.1038/s41598-019-41613-3

**Published:** 2019-03-26

**Authors:** Xiaorong Cheng, Shoulin Gu, Anthony Centeno, Graham Dawson

**Affiliations:** 1 Suzhou Vocational Institute of Industrial Technology, Suzhou, Jiangsu 215104 P. R. China; 2 Jiangsu Key Laboratory of Thin Films and Department of Physics, Soochow University, Suzhou, Jiangsu 215006 P. R. China; 3 Department of Electrical and Electronic Engineering, Xi’an Jiaotong Liverpool University, Suzhou, Jiangsu 215123 P. R. China; 4 Department of Chemistry, Xi’an Jiaotong Liverpool University, Suzhou, Jiangsu 215123 P. R. China

## Abstract

A novel Cu_2_O-Au-BFO heterostructure photocathode was constructed which significantly improved the efficiency of photo-generated carrier transfer for solar hydrogen production. A BiFeO_3_ (BFO) ferroelectric film was synthesized on top of a Cu_2_O layer by a sputtering process. The BFO layer acted to protect the Cu_2_O layer from photochemical corrosion, increasing photoelectrochemical (PEC) stability. The p–n heterojunction between Cu_2_O and BFO layers enhanced the PEC properties by suppressing charge recombination and improved interfacial charge transfer efficiency. When Cu_2_O and BFO are interfaced by Au Nanoparticles (NPs) the PEC performance was further enhanced, due to hot-electron transfer at the plasmonic resonance. After positive poling, the depolarization field across the whole volume of BFO film drove electrons into the electrolyte solution, inducing a significant anodic shift, V_op_ of 1.01 V *vs*. RHE, together with a significantly enhanced photocurrent density of −91 μA/cm^2^ at 0 V *vs*. RHE under 100 mW/cm^2^ illumination. The mechanism was investigated through experimental and theoretivcal calculations.

## Introduction

Harvesting energy from solar power, via photoelectrochemical (PEC) water splitting, is an attractive solution for fulfilling the demand of renewable hydrogen energy^1^. As the most important components of a PEC cell, highly efficient and stable semiconductor photoelectrodes have been extensively studied. Most of the research work has focused on n-type semiconductors as photoanodes, while the reported work on photocathodes is relatively sparse. Here, we report the fabrication and performance of a novel Cu_2_O-Au-BFO photocathode.

Ferroelectric thin films, such as Pb(Zr,Ti)O_3_, BiFeO_3_ (BFO), and BiFeCrO_6_ are a new category of photocathode materials^2,3^. They utilize the depolarization electric field (E_dp_) developed across the whole volume of the film. BFO films have the benefits of being stable in electrolyte, have high open circuit potential (as high as 50 V, which exceeds the bandgap limit totally) and are photo-responsive under visible illumination, with a bandgap of about 2.4 eV^3,4^. Li *et al*. reported that a Bi_2_FeCrO_6_ thin film on SrTiO_3_ substrate photocathode exhibited a high photocurrent of –1.02 mA/cm^2^ at –0.97 V versus reversible hydrogen electrode (*vs*. RHE), and an onset potential (V_op_) of 0.6 V *vs*. RHE^5^. However, the rapid recombination of photo-generated carriers limits the PEC performance.

Heterostructure photoelectrodes, which are comprised of two different semiconductors in stacked layers, have been shown to improve the separation of photo-generated carriers, with the internal electric field in the heterojunction region promoting carrier separation. In a heterostructure, an incremental V_op_ is usually achieved, but the photocurrent is normally limited by the blocked transfer of photo-generated carriers at the interface^6^. The key issue associated with heterostructure photoelectrodes is the energy band edge alignment of the two semiconductor layers. If the energy barrier formed at the interface is too high, the photocurrent is limited^7^. Since BFO is an n-type semiconductor, p-type semiconductor materials with a suitable energy band structure are required to form the heterostructure. Cuprous oxide (Cu_2_O), with a direct bandgap value of 2.0 eV and suitable conduction band level^8^, is a promising p-type material for hydrogen production. The use of Cu_2_O as a photoelectrode for water splitting has been limited due to instability problems upon PEC cycling in aqueous solution. This has required the application of a protective layer which is stable in electrolyte^9^. In this work the BFO ferroelectric film serves additionally as the protective layer in the Cu_2_O-BFO heterostructure, while Cu_2_O forms a heterojunction with BFO for efficient photo-generated carrier separation.

It has been proposed that the localized surface plasmon resonance (LSPR) of noble metal nanoparticles, which are coherent oscillations of their conduction electrons, can be used to enhance the efficiency of photo-generated carriers transfer at the heterostructure interface^10,11^. Noble metal nanoparticles show increased extinction due to LSPR^12,13^, and there has been much work on engineering metal nanoparticles to give LSPRs at wavelengths between the ultraviolet and mid-infrared^14–18^. At the LSPR, there is an enhanced electric field leading to strong light scattering^19^. The interaction of light with the metal particle also leads to the creation of internal fields, causing increased absorption^12,13^. One application where increased absorption efficiency is essential is the enhancement of photocatalysis, or solar energy conversion, by hot electrons^12,20–22^. When a photon excites the LSPR the absorption will yield excited electron-hole pairs which are distributed over a range of energies. Some of these energies will be high enough to tunnel into the vacant states of nearby semiconductors. (A good discussion of the dynamics of plasmons and hot electrons is given by Hartland *et al*.^12^) Injecting hot electrons in to the conduction band of semiconductors directly can prevent the impediment of the interface barrier and improve the energy band edge alignment, as reported in WS_2_-Au-CuInS_2_, ITO-Au-PZT and CdS-Au-SrTiO_3_ photocatalysts^23–26^.

In this work we report, for the first time, on using Gold nanoparticles (Au NPs) at the interface between the Cu_2_O and BFO in a photoelectrode. The LSPR of the Au NPs is utilized to enhance the transfer of photo-generated carriers at the interface. The Cu_2_O-Au-BFO photoelectrode is fabricated using a sputtering and annealing process. The BFO ferroelectric film forms a protective coating and provides the E_dp_ after poling. When the direction of E_dp_ is from the photoelectrode to the electrolyte, the photo-generated electrons can be driven out, resulting in an increase of photocurrent density (−91 μA/cm^2^ at 0 V *vs*. RHE) and V_op_ of 1.01 V *vs*. RHE under 100 mW/cm^2^ illumination in 0.1 M Na_2_SO_4_ solution.

**Figure 1:**
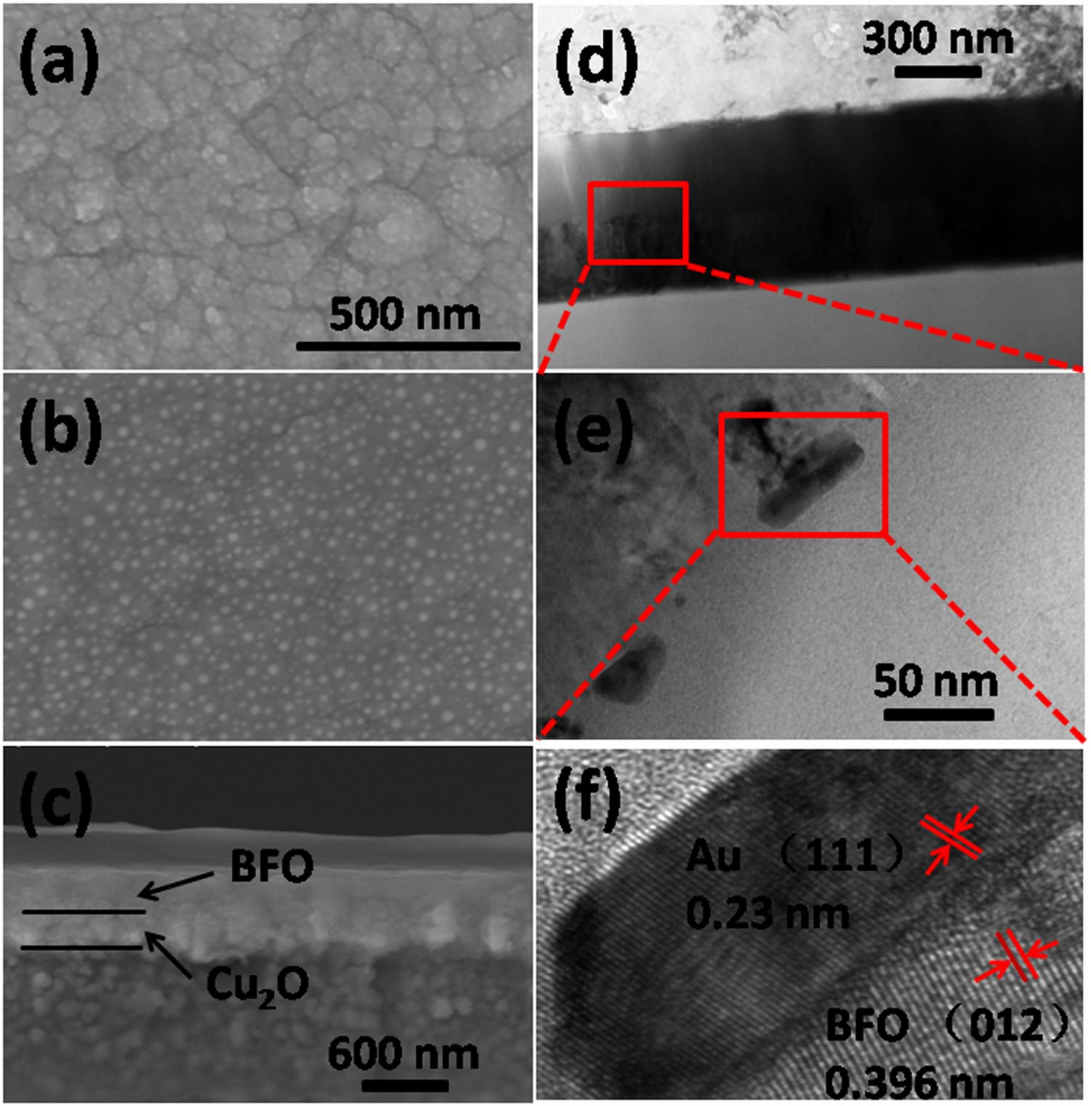
Top-down SEM images of (**a**) Cu_2_O-Au-BFO photoelectrode surface and (**b**) Au NPs on Cu_2_O layer; (**c**) cross-section SEM image of Cu_2_O-Au-BFO photoelectrode on Pt/Ti/SiO_2_/Si(100) substrate; (**d**–**f**) cross-section TEM images of the Cu_2_O-Au-BFO photoelectrode.

The Cu_2_O-Au-BFO photoelectrode was fabricated on Pt/Ti/SiO_2_/Si(100) substrate (Huajing microelectronics Co. Ltd). Firstly, the Cu_2_O layer was deposited on Pt/Ti/SiO_2_/Si(100) substrate by a sputtering process (Cu target, 1 Pa, O_2_:Ar = 1:6, 50 W, 300 °C deposition temperature, sputtering for 30 min). The Au NPs were fabricated on the Cu_2_O layer by a two-step method. Firstly, Au thin films were deposited by sputtering using an Au target under Ar atmosphere (2.5 Pa) for 30 s. Then the sample with the thin Au film was annealed in air at 450 °C for 3 hours. After that, The BFO ferroelectric film was synthesized by sputtering process (1.2 Pa, O_2_:Ar = 12:32, 100 W, 600 °C deposition temperature, sputtering for 1 hour). Figure 1a displays the SEM surface image of Cu_2_O-Au-BFO photoelectrode. This micrograph indicates a well-grown polycrystalline BFO film with a grain size varying from 20 to 200 nm. The surface morphology of the Au NPs deposited on Cu_2_O layer (Fig. 1b) shows that fine Au NPs with high density and good uniformity in distribution have been fabricated by the sputtering and annealing process. The Au NPs are hemi-ellipsoid in shape, with the flat surface on the Cu_2_O layer. The diameter of the two non-bisected axis are seen to be, on average, around 50 nm. The SEM cross-section image of the Cu_2_O-Au-BFO photoelectrode on Pt/Ti/SiO_2_/Si(100) substrate as shown in Fig. 1c illustrates that the thicknesses of Cu_2_O and BFO layers are around 300 nm. The BFO layer covers uniformly on the Cu_2_O layer, which can be clearly observed in the cross-section TEM image of Fig. 1d. Figure 1d shows the overview of the Cu_2_O and BFO layers stacked on the Pt/Ti/SiO_2_/Si(100) substrate successively. The TEM image also confirms the thickness of Cu_2_O and BFO layers are both about 300 nm. The enlarged TEM image in Fig. 1e shows the Au nanoparticles (black particles) between Cu_2_O and BFO layers to be hemi-ellipsoid. The diameters of the non-bisected axis are typically 50 nm although a small number of smaller dimension, down to 20 nm, were also observed. The radius of the bisected axis is ~15 nm.

The (111) planes of Au NPs with lattice spacing of 0.23 nm are shown in the high-resolution TEM image of Fig. 1f, which confirms the constituent of those particles at theCu_2_O/BFO interface, as also observed in SEM and TEM images. The (012) planes of BFO layer with lattice spacing of 0.396 nm are also clearly shown in Fig. 1f, which is in good agreement with a previous report^27^. Figure S1 in Supporting Information shows the energy dispersive spectrometer (EDS) element distribution in the cross-section SEM image, which confirms the components of Cu_2_O-Au-BFO photoelectrode again.

**Figure 2:**
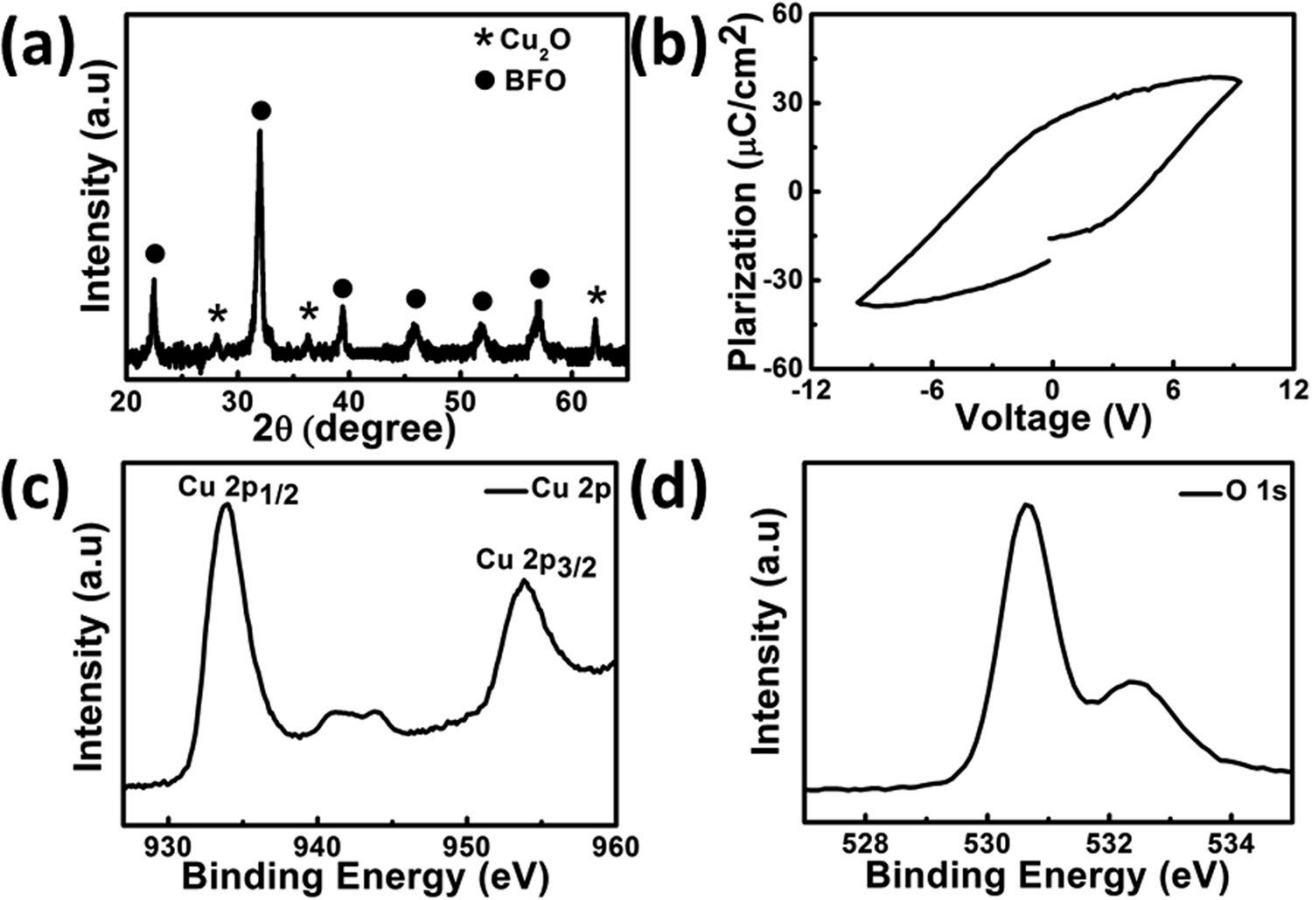
(**a**) XRD pattern of Cu_2_O-Au-BFO photoelectrode; (**b**) PE loop for the BFO film on Pt/Ti/SiO_2_/Si(100) substrate; XPS spectra of (**c**) Cu 2p_1/2_, Cu 2p_3/2_ and (**d**) O 1 s of Cu_2_O in Cu_2_O-Au-BFO photoelectrode.

Figure 2a shows the X-ray diffraction (XRD) pattern of the Cu_2_O-Au-BFO photoelectrode. The diffraction peaks of Cu_2_O can be clearly identified from the pattern, which could be indexed to (110), (111), and (220) planes of cubic Cu_2_O (JCPDS card No. 78–2076). The diffraction peaks of BFO confirm the perovskite crystalline structure (JCPDS card No. 86–1518). However, there is no obvious diffraction peaks of Au NPs observed in the XRD pattern. This is probably due to the small amount of Au in the Cu_2_O-Au-BFO architecture. The Polarization-electric field (PE) loop of the BFO layer is presented in Fig. 2b. As can be seen the BFO layer shows typical ferroelectric hysteresis characteristics. The X-ray photoelectron spectroscopy (XPS) analysis for Cu_2_O layer in Cu_2_O-Au-BFO photoelectrode is performed and presented in Fig. 2c,d, which further confirms the composition of Cu_2_O. The peaks located at 932.1 and 952.0 eV could be ascribed to those of Cu 2P_3/2_ and Cu 2P_1/2_ from Cu_2_O, respectively^28^. The weak peaks around 940.0 eV can be attributed to the small amount of CuO^29^. The O 1 s peak located at 530.8 eV further confirmed the formation of Cu_2_O as shown in Fig. 2d.

**Figure 3:**
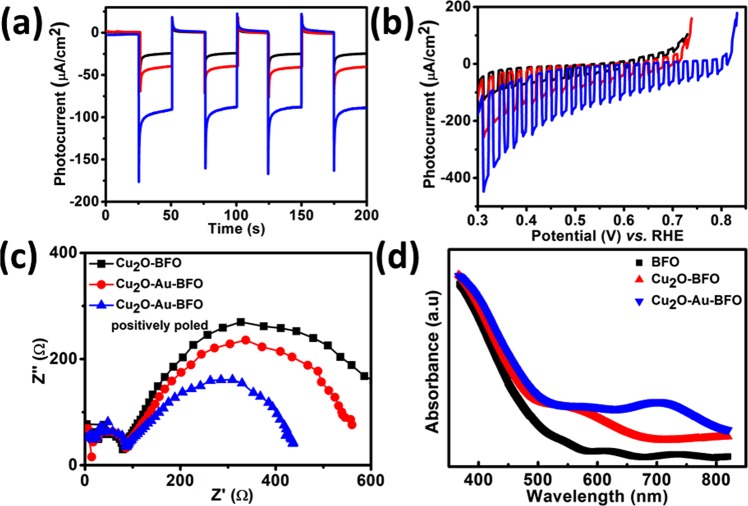
(**a**) J–t and (**b**) J–V curves of photoelectrodes (Cu_2_O-BFO in black, Cu_2_O-Au-BFO in red and Cu_2_O-Au-BFO positively poled in blue); (**c**) EIS spectra of the photoelectrodes; (**d**) UV–vis absorption spectra of the BFO, Cu_2_O-BFO and Cu_2_O-Au-BFO films on quartz substrate.

The poor stability of Cu_2_O in electrolyte leads to a low and declining photocurrent, about −0.05 μA/cm^2^. The BFO photoelectrode exhibits a small anodic photocurrent of about 2.5 μA/cm^2^, which is due to BFO intrinsic n type properties (Fig. S2, ESI†). In Fig. 3a, we present the photocurrent density *vs*. time (J–t) curves for the Cu_2_O-BFO (in black), Cu_2_O-Au-BFO (in red) and Cu_2_O-Au-BFO positively poled (in blue) photoelectrodes. Compared with the Cu_2_O and BFO photoelectrodes, the Cu_2_O-BFO photoelectrode shows an increased photocurrent of around −25 μA/cm^2^, indicating that the p–n heterojunction is beneficial for the separation and transport of photo-generated electron–hole pairs. Incorporating Au NPs into the interface between the Cu_2_O and BFO layers leads to a further 68% increase in photocurrent (−42 μA/cm^2^).

When the BFO ferroelectric film is polarized by a positive applied voltage an opposing depolarization electric field (E_dp_) is established across the whole volume of the BFO layer, that is from the electrolyte to the photoelectrode. The E_dp_ drives the photo-generated charge carriers and enables a more favorable energy level alignment at the Cu_2_O/BFO and photoelectrode/electrolyte interfaces. In this case, the photocurrent increases significantly, to −91μA/cm^2^. Figure 3b presents the photocurrent density *vs*. potential (J–V) curves of different photoelectrodes. As we can see, the V_op_ (where the cathodic photocurrent appears on J–V curves) of Cu_2_O-BFO photoelectrode is 0.71 V *vs*. RHE. For Cu_2_O-Au-BFO, the V_op_ increases to 0.85 V *vs*. RHE and keeps increasing to 1.01 V *vs*. RHE after being positively poled. As expected, the photocurrent decreases to −0.2 μA/cm^2^ and V_op_ decreases to 0.54 V *vs*. RHE if the Cu_2_O-Au-BFO photoelectrode is poled by the reverse electric field (Fig. S3, ESI†). Figure 3c shows the electrochemical impedance spectroscopy (EIS) spectra (Nyquist plot) of the heterostructure photoelectrodes without Au (Cu_2_O-BFO), with Au (Cu_2_O-Au-BFO) and also with poling (Cu_2_O-Au-BFO poled). The introduction of Au NPs lowers the interfacial charge transfer resistance suggesting enhanced charge carrier separation at the interface and, hence, a more efficient PEC efficiency^30^. As can be seen from Fig. 3c positive poling reduced the resistance further. This resultant improvement in PEC activity is shown in Fig. 3a,b.

To further investigate the roles of the Cu_2_O-BFO heterojunction and Au NPs, the UV–vis absorbance spectra of BFO, Cu_2_O-BFO and Cu_2_O-Au-BFO films were measured and are presented in Fig. 3d. Because the Pt/Ti/SiO_2_/Si(100) substrate is too thick and opaque, all the film samples are fabricated on transparent quartz substrates by the same processes. The BFO film displays an absorption edge at around 500 nm, which matches its bandgap (~2.4 eV)^3^. The Cu_2_O-BFO film has two clear edges of BFO and Cu_2_O. The absorption broad peak around 500 to 650 nm comes from the band gap of Cu_2_O (~2.0 eV)^8^. Above the bandgap energy of Cu_2_O, the absorption curve of Cu_2_O-Au-BFO film nearly overlaps that of Cu_2_O-BFO. An enhanced absorption peak centered around 700 nm is observed^11^.

**Figure 4:**
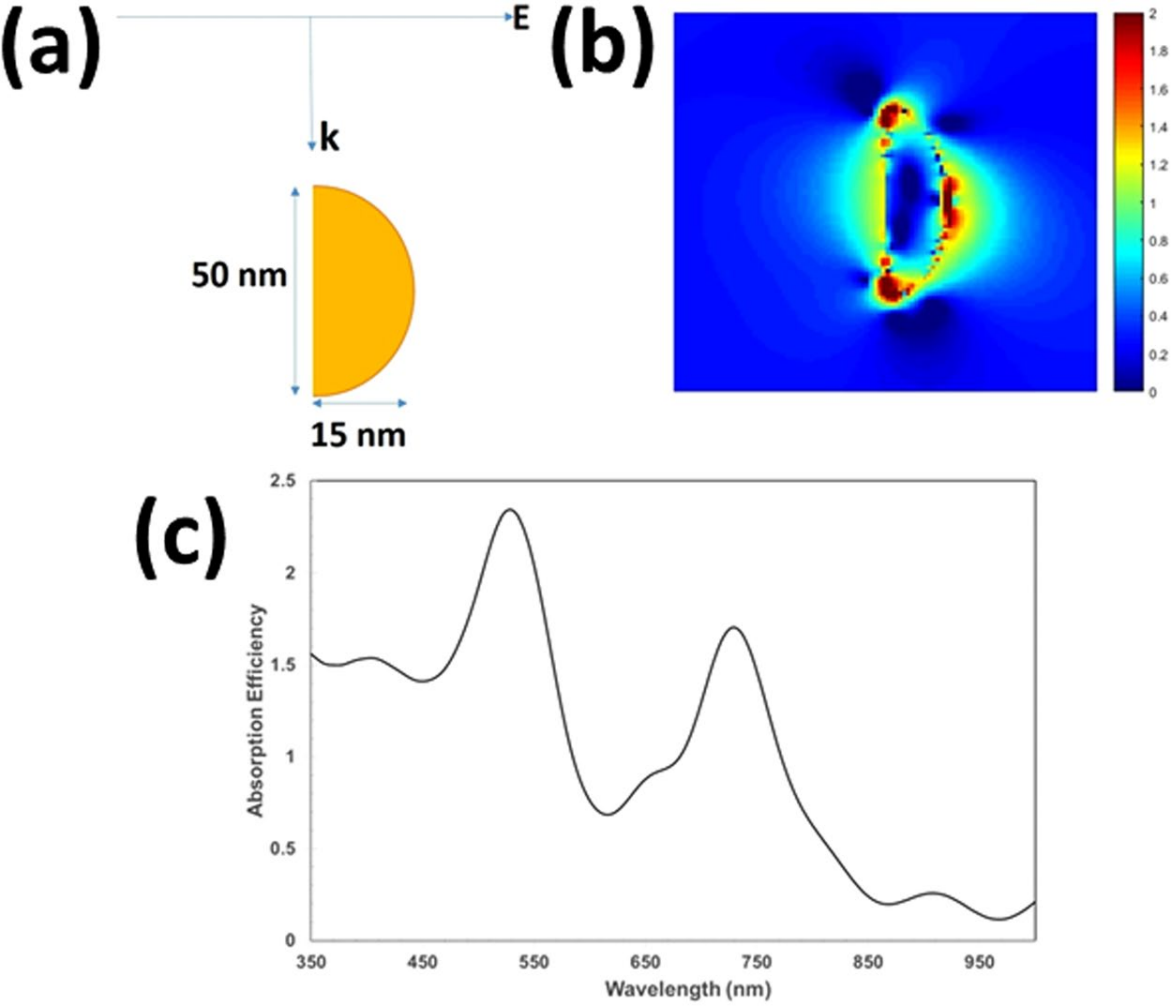
(**a**) Illustration of the FDTD model where the field is incident to the side of the nanoparticle; (**b**) Absorption efficiency of Au nanoparticle; (**c**) Electric field enhancement due to the Au nanoparticle. The electric field enhancement is the square of the normalized electric field, where the normalization is with respect to the field magnitude incident on the particle. It should be noted that the scale is logarithmic.

To confirm that the absorption peak around 700 nm is due to the LSPR of the Au NPs, electromagnetic modelling was carried out, using the Finite Difference Time Domain (FDTD) technique^31^. A full description of the FDTD modelling is given in the supporting information but is briefly described here. The absorption efficiency of Au metal nanoparticles was calculated using the FDTD technique, using the methodology described previously^32^. A number of scenarios were considered in the calculations, but it was found that an incident field on the Au NP, as depicted in Fig. 4a, gave an absorption peak at 727 nm. (That is with light incident on the 15 nm radius dimension of the bisected axis of the hemi-ellipsoid). In this model the refractive index around the nanoparticle was considered to be a single homogenous material, of refractive index 2.55. This was considered to be a reasonable approximation, since there is not a large difference in the refractive index of Cu_2_O (~2.55) and BFO (~2.88). It also enabled the calculation to be carried out with an incident plane wave.

The resulting FDTD calculation of the absorption spectra, Fig. 4b shows an absorption peak at 727 nm, which corresponds closely with that shown in Fig. 3d, for the Cu_2_O-Au-BFO film. Figure 4c shows the calculated Electric field enhancement at 727 nm. It shows a dipolar type mode with peak field enhancement of up to 2 orders of magnitude at electric field “hot-spots” on the surface and just inside the Au particle. Plasmonic electric field hot-spots are known to be important for the process of hot electron generation^12^. This is due to the large enhancement of the electric field and the breaking of the linear momentum of the electron as a result of a strongly non-uniform field^12^. The UV–vis absorbance analysis, along with FDTD calculations, imply that the light-harvesting of Cu_2_O-Au-BFO film extends into the visible light spectra. This in turn leads to an increased photocurrent.

**Figure 5:**
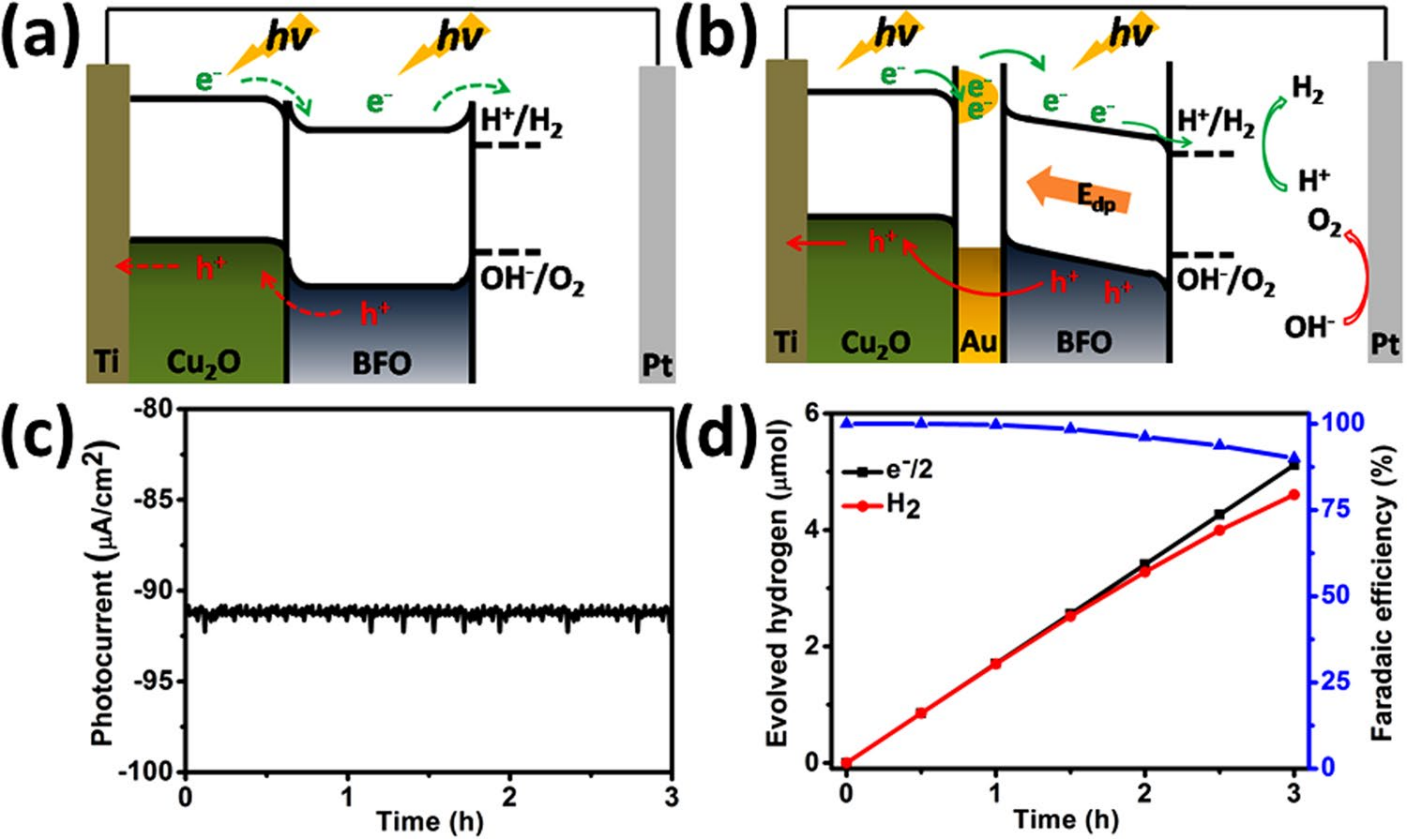
The energy band structure of (**a**) Cu_2_O- BFO photoelectrode and (**b**) Cu_2_O-Au-BFO photoelectrode positively poled; (**c**) dependence of the photocurrent on time and (**d**) hydrogen production on time curves of the Cu_2_O-Au-BFO photoelectrode positively poled.

On the basis of PEC testing, UV-vis results and the FDTD calculations, a physical schematic illustration of the energy band diagrams of the Cu_2_O-BFO photoelectrode and Cu_2_O-Au-BFO photoelectrode, positively poled, are depicted in Fig. 5a,b. These illustrate how the photo-generated charges contribute to the water splitting reaction. It is known that the PEC performance of BFO depends on the fabrication method^33^. The BFO film fabricated by sputtering process exhibits n-type and Cu_2_O exhibits p-type properties in this work, as shown in Fig. S4 in Supporting Information. In our case, Cu_2_O and BFO layer are series stacked and the photo-generated carriers are promptly produced in Cu_2_O and BFO layer under light illumination. The internal electric field induced in the Cu_2_O/BFO heterostructure can contribute to the separation and transport of the photo-generated electron-hole pairs. But this internal electric field only exists in the space-charge region of the Cu_2_O/BFO heterojunction. The energy level drop between the energy bands of Cu_2_O and BFO leads to a potential barrier at the Cu_2_O/BFO interface. Due to the p-type character of BFO, there is an upward energy barrier at the BFO/electrolyte interface, which impedes the photo-generated electrons moving into the electrolyte. If there are no other internal electric fields or effects in the Cu_2_O/BFO electrode, the migration of the photo-generated electrons and holes will not be efficient, which explains the relatively small photocurrent in this case.

When Cu_2_O and BFO layers are interfaced by the Au NPs, a favorable energy level for the separation of photo-generated electron-hole pairs is reached. In this case, the photo-generated electrons from the conduction band of Cu_2_O will migrate to Au NPs and be pumped to the conduction band of BFO by the LSPR hot electron effect, leading to enhanced photocurrent. Furthermore, a depolarizing field (E_dp_) is formed across the whole bulk region of the BFO film. The depolarizing direction is from the electrolyte to the photoclectrode after positive poling, which modifies the potential energy level (i.e. conduction, valence band of BFO and downward barrier at BFO/electrolyte interface) and provides a strong driving force for photo-generated carrier migration. This process is depicted schematically in Fig. 5b. Therefore, more photo-generated electrons and holes can be driven into the electrolyte by E_dp_, leading to enhanced photocurrent and V_op_ anodic shift. Conversely, negative poling will result in decreased photocurrent and V_op_ cathodic shift as shown in Fig. S3, ESI†.

It is important to determine the long-term stability of the Cu_2_O-Au-BFO photoelectrode after positive poling. It can be seen from Fig. 5c that a sustained photocurrent, without obvious decay, is observed during 3 hours of continuous PEC reaction, indicating excellent stability. The corresponding photocurrent density is maintained at −91μA/cm^2^. We believe that the BFO layer prevents the photochemical corrosion of Cu_2_O and also enhances the attachment between the Au NPs and the Cu_2_O, resulting in increased PEC stability. The time course of hydrogen production of positively poled Cu_2_O-Au-BFO photoelectrode is measured and shown in Fig. 5d. The nearly straight line is the theoretical values of hydrogen production calculated based on the electrons passing through the circuit. The water splitting reaction was conducted in 0.1 M Na_2_SO_4_ electrolyte solution with an Ag/AgCl reference electrode and a Pt mesh counter electrode. A 100 mW/cm^2^ Xe lamp is used as a light source and no bias was added between the Cu_2_O-Au-BFO photoelectrode and Pt electrode. During the water splitting reaction, hydrogen bubbles were seen on the surface of the Cu_2_O-Au-BFO photoelectrode and oxygen bubbles observed on the Pt mesh. The amount of evolved hydrogen gas and the photocurrent were both recorded to calculate the Faradic efficiency for hydrogen production. The Faradic efficiency is maintained at nearly 90% within the 3 hour continuous reaction.

In summary, a novel Cu_2_O-Au-BFO photocathode has been fabricated on a Pt/Ti/SiO_2_/Si(100) substrate by a sputtering and annealing process. While a Cu_2_O/BFO heterojunction can effectively improve the separation of photo-generated electrons and holes. The introduction of Au NPs, inserted between the Cu_2_O and BFO layers, leads to an enhanced photocathodic performance. This can be attributed directly to the LSPR of the Au nanoparticle. The E_dp_ in the BFO film also contributes to the photo-generated carriers separation and the removal of the upward potential barrier at the photoelectrode/electrolyte interface. High photocurrent density (−91 μA/cm^2^ at 0 V *vs*. RHE) and V_op_ (1.01 V *vs*. RHE) under 100 mW/cm^2^ illumination are obtained for the Cu_2_O-Au-BFO photocathode. Finally, due to the protection of the BFO layer, the Cu_2_O-Au-BFO photocathode is extremely stable and is a good potential photocathode candidate for water splitting.

## Methods

### Sample characterization

The crystal structures of samples were characterized by measuring X-ray diffraction (XRD) on a Rigaku D/MAX 3 C X-ray diffractometer using CuK_α_ radiation. Surface and cross-section morphology images and energy dispersive spectrometer (EDS) analysis were performed on a Hitachi SU8010 field-emission scanning electron microscope (SEM). X-ray photoelectron spectroscopy (XPS) measurements of Cu_2_O layer was performed at room temperature using a spectrometer hemispherical analyser (ESCALAB 250Xi, Thermo). Transmission electron microscope (TEM) analysis was performed using a Tecnai G220 (S-TWIN, FEI). UV–vis absorption spectra were measured on an Agilent Cary 300 spectrometer. For PEC measurements, all samples were cut into 1.5 × 1.5 cm^2^ segments. Tinned copper wire was connected to the Pt layer of Pt/Ti/SiO_2_/Si(100) substrate by gallium-indium eutectic (Sigma-Aldrich). The exposed backside and edges were sealed with an industrial epoxy (PKM12C-1, Pattex). The photocurrent-time (J-t) and photocurrent-potential (J-V) curves were measured by an electrochemical workstation (CHI660D, CH Instrument) with a 100 mW/cm^2^ Xe lamp (Oriel, Newport Co.) as light source and 0.1 M Na_2_SO_4_ solution as electrolyte (pH = 7). For the PEC measurement, samples served as the working electrode, a Pt wire as the counter electrode and an Ag/AgCl electrode as the reference electrode. The potentials were re-scaled to *vs*. RHE according to the following equation:

$${{\rm{E}}}_{({\rm{RHE}})}={\rm{E}}+{{\rm{E}}}_{({\rm{Ag}}/{\rm{AgCl}})}+0.059\times {\rm{pH}}$$

where E_(Ag/AgCl)_ = 0.197 V.

The electrochemical impedance spectroscopy (EIS) and Mott–Schottky plot were also measured by electrochemical workstation in 0.1 M Na_2_SO_4_ electrolyte without illumination. In order to measure the ferroelectric properties of BFO film, a BFO layer with the same thickness and fabrication method as Cu_2_O-Au-BFO was deposited on Pt/Ti/SiO_2_/Si(100) substrate and Pt top electrodes with diameters of 0.28 mm were sputtered onto the BFO layer. The Polarization-electric field (PE) loop was examined using radiant precision ferroelectric analyser (Radiant Technology Co.).

### Poling the BFO films

For poling the ferroelectric BFO films, a pulsed potential of +10 V or −10V with an alternating 0.2 s interval time was applied between sample and the Ag/AgCl reference electrode by an electrochemical workstation (CHI660D) in 0.1 M Na_2_SO_4_ electrolyte.

### Measurement of hydrogen evolution

Hydrogen evolution was measured using a homemade quartz glass air-tight photo-reactor. During measurement, the sample photoelectrode and a Pt mesh were placed in two different tubular chambers. This ensured that the generated hydrogen and oxygen was separated into two chambers. The water splitting reaction was conducted in 0.1 M Na_2_SO_4_ electrolyte under 100 mW/cm^2^ Xe lamp illumination. The amount of produced hydrogen was determined by gas chromatography (Tianmei, GC 7890 T). During the water splitting reaction, the electric charge passing through the circuit is found by integrating the photocurrent in the electrochemical workstation. By dividing the total quantity of charge evaluated by the elementary charge (1.60217662 × 10^19^ C), the amount of electrons (A_e_) taking part in the water splitting reaction is found. Under ideal conditions, two electrons passing through the circuit would convert into one hydrogen molecule, so the Faradic efficiency (E_F_) can be calculated by the following equation:

$${{\rm{E}}}_{{\rm{F}}}=\frac{2{{\rm{A}}}_{{\rm{h}}}{{\rm{N}}}_{{\rm{A}}}}{{{\rm{A}}}_{{\rm{e}}}}\times 100 \% $$

Where N_A_ is Avogadro’s constant (6.022 × 10^23^); A_h_ is the amount of hydrogen.

## Supplementary information

Plasmonic enhanced Cu2O-Au-BFO photocathodes for solar hydrogen production

## Data Availability

All data supporting this study are provided as supplementary information accompanying this paper.

## References

[1] Varadhan P (2017). Surface Passivation of GaN Nanowires for Enhanced Photoelectrochemical Water-Splitting. Nano Lett..

[2] Cheng XR (2014). The photocathodic properties of a Pb(Zr_0.2_Ti_0.8_)O_3_ wrapped CaFe_2_O_4_ layer on ITO coated quartz for water splitting. Chem. Commun..

[3] Liu Q (2016). Enhanced ferroelectric photoelectrochemical properties of polycrystalline BiFeO_3_ film by decorating with Ag nanoparticles. Appl. Phys. Lett..

[4] Bhatnager A, Chaudhuri AR, Kim YH, Hesse D, Alexe M (2013). Role of domain walls in the abnormal photovoltaic effect in BiFeO_3_. Nat. Commun..

[5] Li S (2015). Epitaxial Bi_2_FeCrO_6_ multiferroic thin film as a new visible light absorbing photocathode material. Small.

[6] Cheng XR, Dong W, Zheng FG, Fang L, Shen MR (2015). Enhanced photocathodic behaviors of Pb(Zr_0.20_Ti_0.80_)O_3_ films on Si substrates for hydrogen production. Appl. Phys. Lett..

[7] Shaner MR (2014). Photoelectrochemistry of core–shell tandem junction n–p_+_-Si/n-WO_3_ microwire array photoelectrodes. Energy Environ. Sci..

[8] Hou JG (2014). High-performance p-Cu_2_O/n-TaON heterojunction nanorod photoanodes passivated with an ultrathin carbon sheath for photoelectrochemical water splitting. Energy Environ. Sci..

[9] Liu LM (2014). Synthesis of Cu_2_O nanospheres decorated with TiO_2_ nanoislands, their enhanced photoactivity and stability under visible light illumination, and their post-illumination catalytic memory. ACS Appl. Mater. Interfaces.

[10] Linic S, Christopher P, Ingram DB (2011). Plasmonic-metal nanostructures for efficient conversion of solar to chemical energy. Nat. Mater..

[11] Pu YC (2013). Au nanostructure-decorated TiO_2_ nanowires exhibiting photoactivity across entire UV-visible region for photoelectrochemical water splitting. Nano Lett..

[12] Hartland GV, Besteiro LV, Johns P, Govorov AO (2017). What’s so hot about electrons in metal nanoparticles?. ACS Energy Lett..

[13] Maier, S. A. Plamonics: Fundamentals and applications. *Springer Science and Business Media: New York* (2007).

[14] Jensen TR, Malinsky MD, Haynes CL, Van Duyne RP (2000). Nanosphere Lithography:  Tunable localized surface plasmon resonance spectra of silver nanoparticles. J. Phys. Chem. B.

[15] Theodorou IG (2017). Gold nanostar substrates for metal-enhanced fluorescence through the first and second near-infrared windows. Chem. of Mats..

[16] Xie F (2013). Nanoscale control of Ag nanostructures for plasmonic fluorescence enhancement of near-infrared dyes. Nano Research.

[17] Knight MW (2014). ACS Nano.

[18] Atwater HA, Polman A (2010). Plasmonics for improved photovoltaic devices. Nat. Mater..

[19] Sonnichsen C (2002). Drastic reduction of plasmon damping in gold nanorods. Phy. Rev. Lett..

[20] Clavero C (2014). Plasmon-induced hot-electron generation at the nanoparticle/metal-oxide interfaces for photovoltaic and photocatalytic devices. Nat. Photonics.

[21] Moskovits M (2015). The case for plasmon-derived hot carrier devices. Nat. Nanotechnol..

[22] Narang P, Sundararaman R, Atwater HA (2016). Hot carrier dynamics in solid-state and chemical systems for energy conversion. Nanophotonics.

[23] Yu SJ, Kim YH, Lee SY, Song HD, Yi J (2014). Hot-electron-transfer enhancement for the efficient energy conversion of visible light. Angew. Chem. Int. Ed..

[24] Zhong YQ (2014). Plasmon-assisted water splitting using two sides of the same SrTiO_3_ single-crystal substrate: conversion of visible light to chemical energy. Angew. Chem. Int. Ed..

[25] Cheng ZZ (2015). Au plasmonics in a WS_2_-Au-CuInS_2_ photocatalyst for significantly enhanced hydrogen generation. Appl. Phys. Lett..

[26] Wang ZJ (2016). Manipulation of charge transfer and transport in plasmonic-ferroelectric hybrids for photoelectrochemical applications. Nat. Commun..

[27] Hu ZJ (2017). Facile synthesis of Sm-doped BiFeO_3_ nanoparticles for enhanced visible light photocatalytic performance. Materials Science and Engineering B.

[28] Zhu H (2013). A new strategy for the surface-free-energy-distribution induced selective growth and controlled formation of Cu_2_O–Au hierarchical heterostructures with a series of morphological evolutions. J. Mater. Chem. A.

[29] Poulston S, Parlett PM, Stone P, Bowker M (1996). Surface oxidation and reduction of CuO and Cu_2_O studied using XPS and XAES. Surf. Interface. Anal..

[30] Lopes T, Andrade L, Ribeiro HA, Mendes A (2010). Characterization of photoelectrochemical cells for water splitting by electrochemical impedance spectroscopy. Int. J. Hydrogen Energy.

[31] Oskooi AF (2010). MEEP: a flexible free-software package for electromagnetic simulations by the FDTD method. Compuy. Phy. Commun..

[32] Centeno A, Ahmed B, Reehal H, Xie F (2013). Diffuse scattering from hemispherical nanoparticles at the air-silicon interface. Nanotechnology.

[33] Maso′ N, West AR (2012). Electrical properties of Ca-doped BiFeO_3_ ceramics: from p-type semiconduction to oxide-ion conduction. Chem. Mater..

