# Supplementary material for: Plasmonic enhanced Cu2O-Au-BFO photocathodes for solar hydrogen production
Source: Sci Rep. 2019 Mar 26;9:5140. doi: 10.1038/s41598-019-41613-3 (PMC6435794; doi:10.1038/s41598-019-41613-3)
Supplement: Supplementary file 1 — Plasmonic enhanced Cu2O-Au-BFO photocathodes for solar hydrogen production [file 41598_2019_41613_MOESM1_ESM.pdf]

Electronic Supporting Information

Plasmonic enhanced Cu_2O-Au-BFO photocathodes for solar hydrogen production

Xiaorong Cheng*[1], Shoulin Gu[2], Anthony Centeno[3] and Graham Dawson[4]

[1.] Suzhou Vocational Institute of Industrial Technology, Suzhou, Jiangsu 215104, P. R. China

[2.] Jiangsu Key Laboratory of Thin Films and Department of Physics, Soochow University, Suzhou, Jiangsu 215006, P. R. China

[3.] Department of Electrical and Electronic Engineering, Xi'an Jiaotong Liverpool University, Suzhou, Jiangsu 215123, P. R. China

[4.] Department of Chemistry, Xi'an Jiaotong Liverpool University, Suzhou, Jiangsu 215123, P. R. China

* xiaorongcheng@126.com

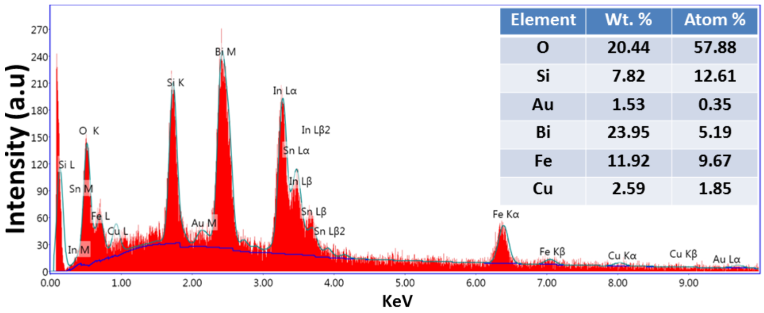

Fig. S1 EDS analysis of the cross-section Cu_2O-Au-BFO photoelectrode.

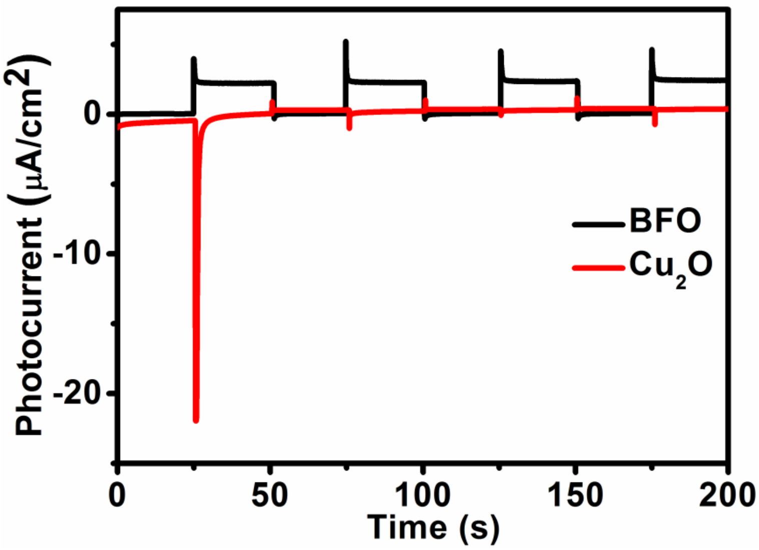

Fig. S2 J–t curves of BFO and Cu_2O photoelectrodes.

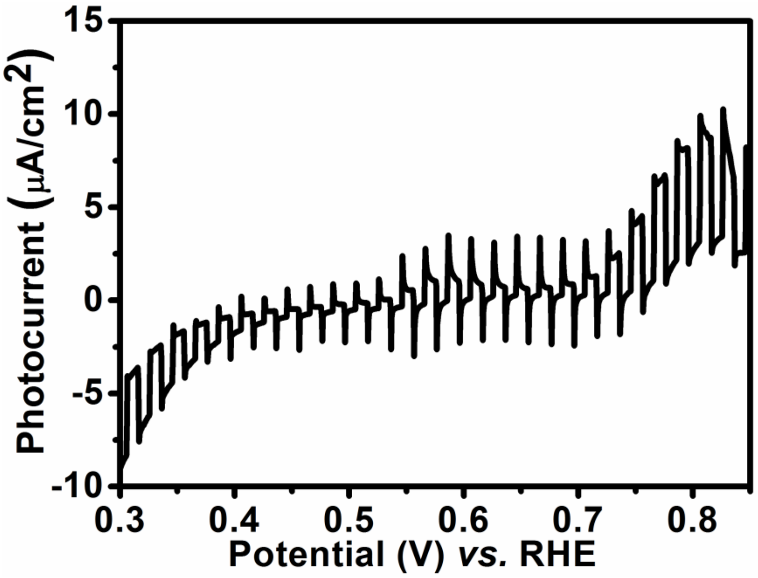

Fig. S3 J–V curve of Cu_2O-Au-BFO photoelectrode negatively poled.

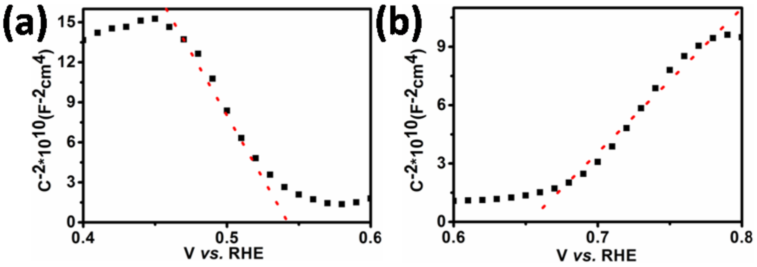

Fig. S4 Mott-Schottky plots of (a) Cu_2O and (b) BFO.

Mott–Schottky measurements are performed on 300 nm Cu_2O and BFO layer deposited on FTO glass substrate by the same fabrication process described in experimental section, respectively. The capacitance (C) as a function of the applied potential (V) are shown in Fig. S4. The negative slope of $1/C^2$ *vs.* V demonstrates p-type character of Cu_2O film. And the positive slope demonstrates n-type character of BFO film.[1]

Finite Difference Time Domain Calculations

Introduction

The aim of these calculations was to investigate the increased absorption due to Au nanoparticles in the Cu_2O-Au-BFO system at ~700 nm. The Au nanoparticles are assumed to have the dimensions illustrated in Figure S1. It is also considered that the absorbance peak at 700 nm is caused by a Localized Surface Plasmon Resonance (LSPR) of the Au nanoparticle.

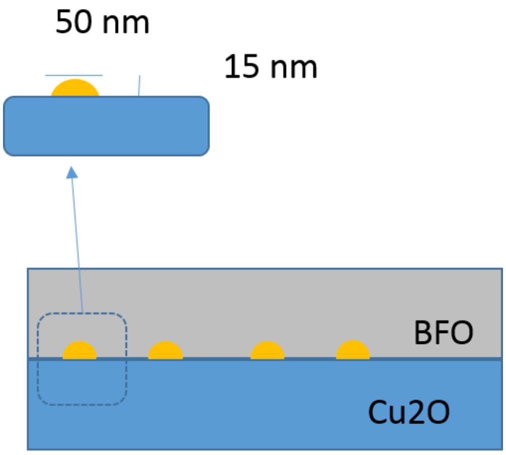

Fig. S5. Dimensions and position of Au nanoparticle in Cu_2O-Au-BFO film

The absorption cross-section, σ_{abs}, of an object much smaller than the wavelength, is defined as the power dissipated in the object under plane wave illumination, divided by the power density of the incident wave on the cross-sectional area, Si. Similarly the scattering cross section, σ_{sca}, is defined by considering the power scattered from the object. If we consider a surface totally enclosing the scattering object then we can calculate the normalized absorption (Q_{abs}) and scattering (Q_{sca}) efficiencies from reference:[2]

$$Q_{abs} = \frac{\sigma_{abs}}{A} \tag{1}$$

$$Q_{abs} = \frac{\sigma_{abs}}{A} \tag{2}$$

Where A is the cross-sectional area and:

$$\sigma_{abs} = \frac{\int_s \mathrm{Re}\left(\frac{1}{2}\vec{E}\times\vec{H}^*\right)d\vec{a}}{|S_i|} \tag{3}$$

$$\sigma_{sca} = \frac{-\int_s \mathrm{Re}\left(\frac{1}{2}\vec{E}_s\times\vec{H}_s^*\right)d\vec{a}}{|S_i|} \tag{4}$$

In equations (4) the subscript means that the scattered field, rather than the total field is considered. The integral is over a surface that fully encloses the Au nanoparticle.

In the FDTD model the Au was modelled using a Drude-Lorentz model with one Drude and five Lorentz terms in the summation:[3]

$$\varepsilon(\omega) = \varepsilon_\infty + \sum_{i=1}^{n} \frac{\alpha_i \omega_p^2}{\omega_{oi}^2 - \omega^2 - j\omega\tau_i} \tag{1}$$

where ω_p is the plasma frequency, α is the strength of the oscillators, ω_o is the resonant frequency of each oscillator, j is the imaginary unit and τ is the damping frequency of each oscillator. Table 1 gives the values that were used.

Table 1. Parameters used in the Drude-Lorentz Model

Gold ω_p=9.03 eV						
i	1	2	3	4	5	6
α	0.760	0.024	0.010	0.071	0.601	4.384
τ (eV)	0.053	0.241	0.345	0.870	2.494	2.214
ω_o(eV)	0.000	0.415	0.830	2.969	4.304	13.32

The open source FDTD software MEEP was used to run the electromagnetic models.[4] Simulations were carried out on a 2*12 core node (Xeon E5-2692V2) processor with 64GB of memory. The spatial resolution of the FDTD model was 1 nm and the workspace was terminated using perfectly matched layers.

Initially, the field was considered to be an incident plane wave on the upper surface (the top of the dome) of the nanoparticle, as illustrated in Fig. S6. The result obtained for the absorption efficiency is shown in Figure 3. In this model the Cu_2O and BFO are considered to have a refractive indexes of 2.5 and 2.88, respectively.

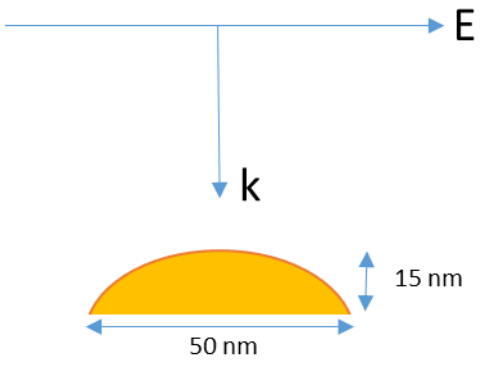

Fig. S6. Model used for initial FDTD calculations of absorption efficiency.

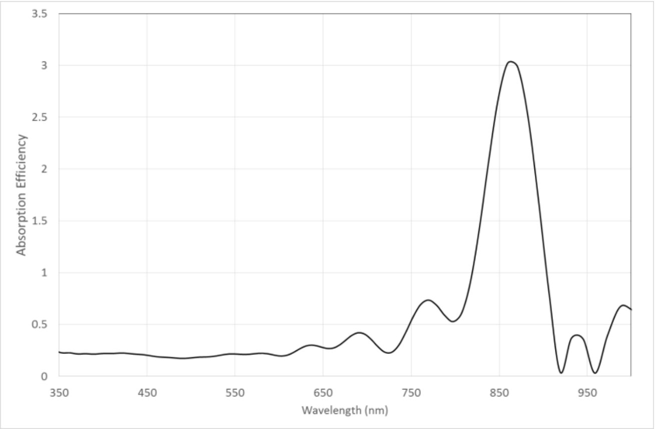

Fig. S7. Absorption efficiency calculated using FDTD for an isolated Au particle on Cu_2O and surrounded by BFO. Fig. S7 shows an LSPR peak at 859 nm, which is a significantly longer wavelength than the absorption peak obtained by UV-Vis measurement for the Cu_2O-Au-BFO film (Fig. 3d in main paper). Due to the incident angle of

the field this LSPR peak is considered to be due to the 50 nm diameter of the semi-ellipsoid particle. The other dimension is 15 nm, so the FDTD model was modified to consider a field incident from the side, as shown in Fig. S8.

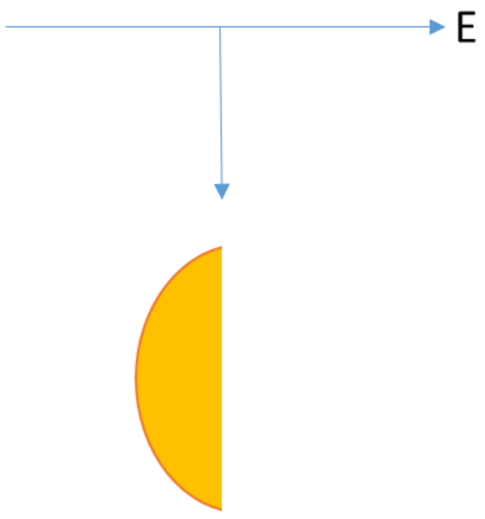

Fig. S8. Illustration of the FDTD model where the field is incident to the side of the nanoparticle.

In this model the refractive index around the nanoparticle was considered to be a single material of refractive index 2.55. This was considered to be a reasonable approximation since there is not a large difference in the refractive index of Cu_2O and BFO, and it enabled a plane wave to be incident on the particle. The result of the calculation using FDTD is shown in Fig. S9.

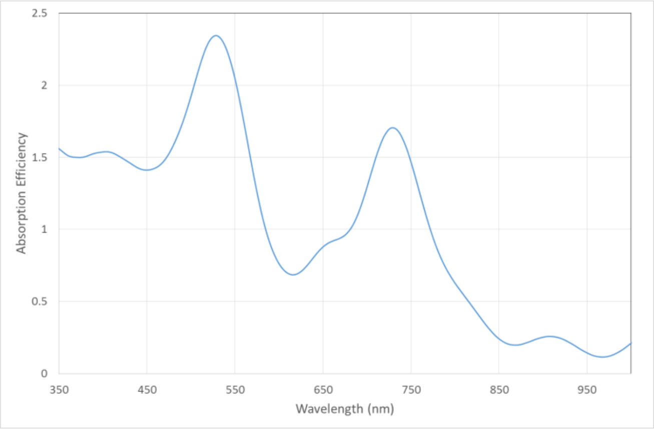

Fig. S9. Absorption efficiency of Au nanoparticle with field incident from the side, as shown in Figure 5.

Fig. S9 shows an absorption peak at 727 nm, which corresponds closely with the absorption peak shown in Fig. 3d, for the Cu_2O-Au-BFO film. Fig. S10 shows the Electric field enhancement calculated, using FDTD, at 727 nm. The result show a dipolar type mode with peak field enhancement of up to 2 orders of magnitude at the surface of the Au.

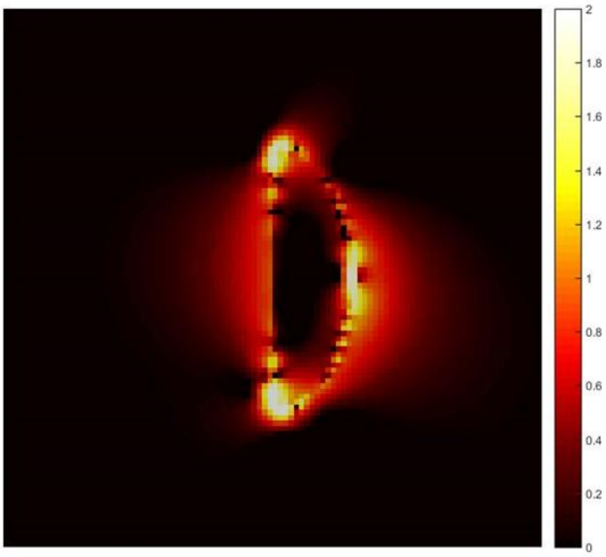

Fig. S10. Electric field enhancement due to the Au nanoparticle. The electric field enhancement is the square of the normalized electric field, where the normalization is with respect to the field magnitude incident on the particle. It should be noted that the scale is logarithmic.

To consider this further the case where the incident field is at 45 degrees, so there are components of field incident on the top (dome) and the side of the Au was considered. The calculated absorption efficiency is shown in Fig. S11, and the electric field enhancement in Fig. S12. It should be noted here that it is actually easier to "tilt" the particle in the FDTD model as incident fields with obtuse (non-normal) angle of incidence do not have a constant incident angle with frequency.

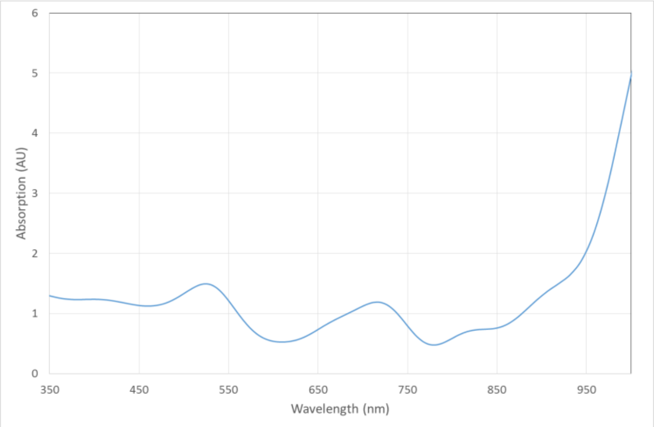

Fig. S11. Absorption due to 45 degree incident field. (Note the results have not been normalized to cross-sectional area hence arbitrary units).

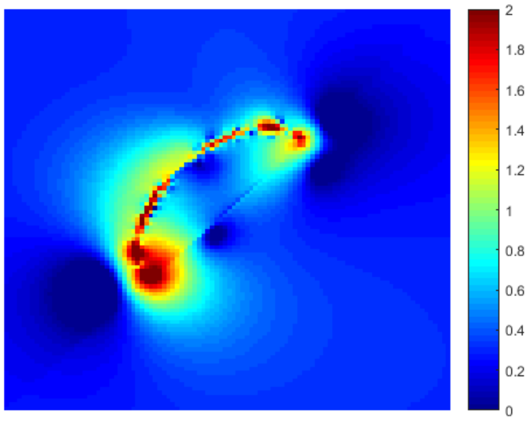

Fig. S12. Electric Field enhancement for an incident field of 45 degrees at 712 nm. (In the FDTD model the particle was actually rotated 45 degrees and the incident field was from the top.)

Fig. S11 shows an absorption peak at 712 nm, with again corresponds closely with the absorption maxima for the Cu_2O-Au-BFO film. Fig. S12 shows that there is field enhancement up to 2 orders of magnitude at this wavelength.

The FDTD analysis infers that the Au nanoparticle of dimensions shown in Fig. S5, with a field component incident on the 'side' of the particle, would have an absorption peak that corresponds to that of the Cu_2O-Au-BFO film. The field enhancement is around two orders of magnitude at various points on the surface.

EIS Analysis

The EIS Nyquist plot was fitted to an equivalent circuit consisting of two RC parallel networks. This was accomplished using the free EIS spectrum analyzer software (**http://www.abc.chemistry.bsu.by/vi/analyser/**). Figures S13-S15 show the results for the three photoelectrodes where the green line is the fitting and the red points the measurements. The low frequency impedance value is R1+R2, where R2 is the charge transfer resistance in the PEC reaction.

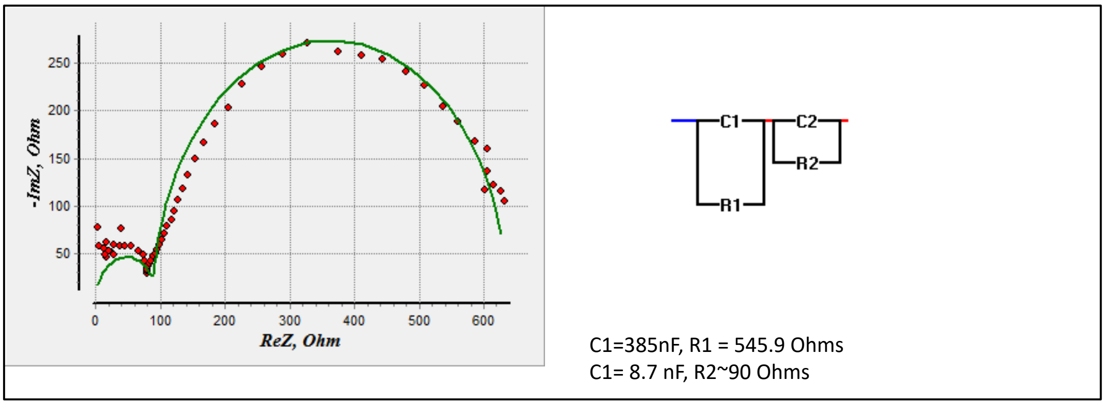

Fig. S13. Cu_2O-BFO

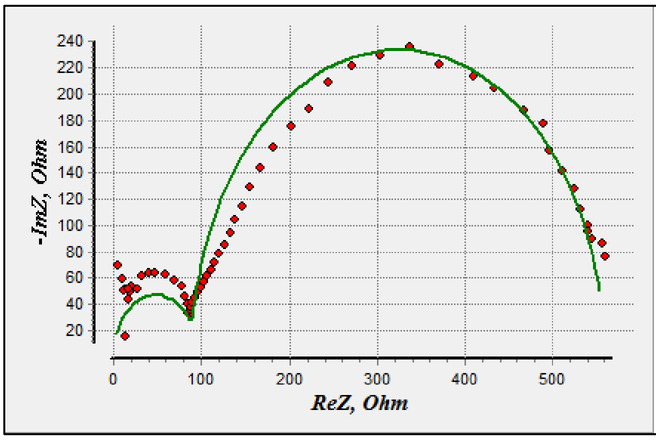

C1=366nF, R1 = 468 Ohms
C1= 8.9 nF, R2~90 Ohms

Fig. S14. Cu_2O-Au-BFO

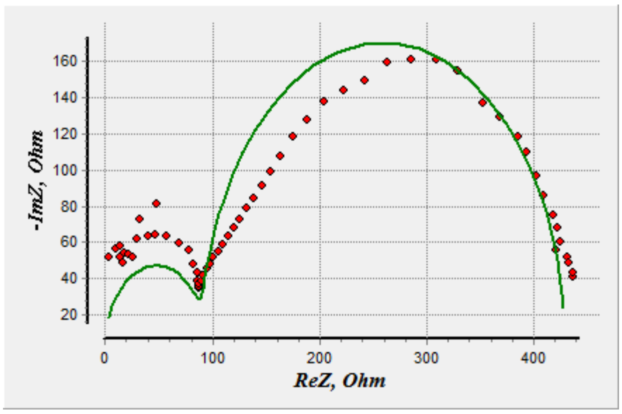

C1=330nF, R1 = 339.9 Ohms
C1= 8.4 nF, R2~90 Ohms

Fig. S15. Cu_2O-Au-BFO (Poled)

The high frequency fit is not so good for the Au structures. We attribute this to the charge transfer across the heterojunction. Nevertheless, since the low frequency curve fit is quite good in all cases, illustrating that the Au particles at the heterojunction cause a reduction in charge transfer resistance at the BFO-electrolyte interface.

References:

1 Koffyberg, F. P., & Benko, F. A. A photoelectrochemical determination of the position of the conduction and valence band edges of p‐type CuO. *J. Appl. Phys.* **3**, 1173-1177 (1982).

2 Centeno, A., Ahmed, B., Reehal, H. & Xie, F. Diffuse scattering from hemispherical nanoparticles at the air-silicon interface. *Nanotechnology* **24**, 415402 (2013).

3 Rakic, A. D., Djurisic, A. B., Elavar, J. M. & Majewski, M. L. Optical properties of metallic films for vertical-cavity optoelectronic devices. *Appl. Opt.* **37**, 5271 (1998).

4 Oskooi, A. F., Roundy, D., Ibanescu, M., Bermel, P., Joannopoulous, J. D. & Johnson, S. G. MEEP: a flexible free-software package for electromagnetic simulations by the FDTD method. *Compuy. Phy. Commun.* **181**, 687-702 (2010).
